# Mg/S@g-C_3_N_4_ nanosheets: A promising fluorescence sensor for selective Cu^2+^ detection in water

**DOI:** 10.1016/j.heliyon.2024.e31785

**Published:** 2024-05-23

**Authors:** Z.A. Alrowaili, Asmaa I. El-Tantawy, S.A. Saad, M.H. Mahmoud, Karam S. El-Nasser, Taha Abdel Mohaymen Taha

**Affiliations:** a Physics Department, College of Science, Jouf University, P.O. Box 2014, Sakaka, Saudi Arabia; b Physics and Engineering Mathematics Department, Faculty of Electronic Engineering, Menoufia University, Menouf 32952, Egypt; c Engineering Basic Sciences Department, Faculty of Engineering, Menoufia University, Shibin al-Kawm, Egypt; d Physics Department, College of Science and Arts, Jouf University, P.O. Box 756, Al-Gurayyat, Saudi Arabia; e Chemistry Department, College of Science and Arts, Jouf University, P.O. Box 756, Al-Gurayyat, Saudi Arabia

**Keywords:** Mg/S@g-C_3_N_4_, Fluorescence sensor, Heavy metal, Band gap, Nanosheet

## Abstract

This work describes the development of a novel fluorescence sensor based on magnesium/S@g-C_3_N_4_ nanosheets for selective detection of copper (Cu^2+^) ions in water. Mg/S@g-C_3_N_4_ nanosheets were prepared by the polycondensation technique and investigated by X-ray diffraction (XRD), ATR-FTIR spectroscopy, scanning electron microscopy (SEM), surface area (BET), and UV–Vis optical absorption measurements. XRD and ATR-FTIR analysis showed the characteristic peaks for S@g-C_3_N_4_. The broad full width at half maximum (0.056 radians) implies a smaller crystallite size, representing smaller Mg/S@g-C_3_N_4_ sheets. SEM micrograph showed non-exfoliated nanosheets with flake-like structures. The EDS mapping confirmed the presence of magnesium, carbon, nitrogen, and sulfur throughout the nanosheets. The Mg/S@g-C_3_N_4_ nanosheets possess a high surface area of 40 m^2^/g and mesopores within the nanosheets, with a size of 1.57 nm. The band gap of the Mg/S@g-C_3_N_4_ nanosheet was estimated to be 3.0 eV. The sensor exhibits a strong quenching response towards Cu^2+^ ions, with a decrease in fluorescence intensity as the concentration of Cu^2+^ increased from 1 μM to 20 μM. The Stern-Volmer quenching constant (K_SV_) showed a relatively high value of 185053 M^−1^. The estimated value of LOD by the Mg/S@g-C_3_N_4_ sensor for Cu^2+^ was 16.2 nM. The sensor offered high sensitivity and selectivity for Cu^2+^ detection over other heavy metals.

## 1 Introduction

Heavy metals are naturally occurring elements with high atomic masses and densities. While some are essential for biological processes in very minute quantities, many are highly toxic, even at low concentrations. These metals can accumulate in the body over time, leading to a variety of health problems [[1], [2], [3]]. The misconception that "pure" water is inherently safe is a major reason why heavy metal detection is crucial. Natural sources like springs and wells, as well as processed drinking water, can become contaminated through natural geological processes, industrial activities, and aging infrastructure [[4], [5], [6]]. By detecting the presence and concentration of heavy metals, potential health risks can be identified before they cause harm. Based on the detected levels, appropriate actions can be taken, such as installing water filters, seeking alternative water sources, or implementing remediation measures. By ensuring water quality meets established safety standards, communities can be protected from the harmful effects of heavy metal exposure [7,8].

Various sophisticated sensing technologies have emerged as guardians of water purity, playing a crucial role in detecting these harmful elements. Atomic absorption spectroscopy (AAS) uses light absorption to identify specific elements, including heavy metals [9]. However, AAS requires complex instrumentation and skilled personnel, making it less suitable for real-time monitoring or field applications. Inductively coupled plasma mass spectrometry (ICP-MS) offers exceptional detection limits for various elements [10]. However, ICP-MS is expensive, bulky, and requires specialized laboratories, limiting its use for on-site monitoring. Electrochemical sensors rely on electrical responses to detect specific ions, including heavy metals [11]. While offering advantages like portability and real-time monitoring, they may suffer from interference from other ions present in water. While traditional methods have their merits, fluorescence sensors offer a compelling alternative for detecting heavy metals in pure water. Their high sensitivity, selectivity, real-time monitoring capabilities, and user-friendliness make them ideal for ensuring water quality in diverse settings, from laboratory settings to remote locations. As research in fluorescence sensor technology continues, their potential for accurate, efficient, and cost-effective heavy metal detection will likely see further advancements [12].

Researchers are constantly striving to develop sensors with high selectivity for specific heavy metals. This is crucial because water samples often contain a mixture of different metals. By minimizing interference from other elements, researchers can create more accurate and reliable sensors. There's a growing exploration of new fluorescent materials for sensor development. These materials offer unique properties like tunable emission wavelengths and enhanced interaction with specific metals. P. Keerthana et al. [13] developed a pyrene carboxaldehyde-carbon quantum dots (PC-CD) fluorescent nanosensor for detecting cadmium (Cd^2+^) ions. The sensor demonstrated excellent performance in a concentration range of 0–70 μM for Cd^2+^ detection. Moreover, the sensor has a low detection limit of 15 nM, indicating its sensitivity for even small amounts of Cd^2+^. The study highlighted the sensor's high selectivity for Cd^2+^ over other metal ions. Samarjit Pattnayak et al. [14] described a novel method for detecting lead (Pb^2+^) ions using l-Glutathione (GSH) modified graphitic carbon nitride quantum dots (GSH@g-C_3_N_4_ QDs). The fluorescence quenching follows a linear relationship with Pb^2+^ concentration within the range of 0.01 μM–0.1 μM. This allows for accurate quantification of Pb^2+^ within this range. The sensor has a low limit of detection (LOD) of 0.025 μM, meaning it can detect very low concentrations of Pb^2+^ ions. The sensor offered high sensitivity, selectivity, and stability. Another study demonstrated the development of a promising Cr^6+^ sensor using novel carbon dots mixed graphene quantum dots (CDs@GQDs) nanohybrid material [15]. The results showed high selectivity for Cr^6+^ detection through fluorescence quenching. The limit of detection (LOD) and limit of quantification (LOQ) were found to be 17.2 nM and 52.2 nM, respectively. Another study highlighted the potential of Zn_3_N_2_ QDs as a simple, efficient, and selective sensor for detecting Cu^2+^ and Mn^2+^ ions in environmental samples [16]. The quenching effect is linear within specific concentration ranges for both Cu^2+^ (2.5–50 μM) and Cu^2+^ (0.05–5 μM). The detection limits of 21.77 nM for Cu^2+^ and 63.82 nM for Mn^2+^ were impressive, indicating the sensor's sensitivity to even small amounts of these metal ions.

Graphitic carbon nitride (g-C_3_N_4_) has unique combination of fluorescence properties, stability, and ease of synthesis, presents a promising avenue for developing efficient and cost-effective sensors for detecting heavy metal contamination [17,18]. X. Zhang et al. [19] showed that W18O49 nanobelts grown on crystalline g-C_3_N_4_ (W18O49–CCN) formed better interfaces compared to those grown on amorphous g-C_3_N_4_ (W18O49-ACN). W18O49–CCN exhibited significantly higher photogenerated charge carrier separation and transport efficiency. This resulted in a much higher H_2_O_2_ evolution rate compared to both pristine crystalline g-C_3_N_4_ and W18O49-CAN. Moreover, showed an effective NO removal with good NO_2_ selectivity. Another study showed that the presence of the carbon layer on the Co nanoparticles facilitates the movement of electrons, leading to significantly improved photocatalytic activity for H_2_O_2_ generation and NO oxidation compared to pristine g-C_3_N_4_ [20]. The optimized composite material showed an impressive 1000-fold increase in H_2_O_2_ generation efficiency compared to pure g-C_3_N_4_ under visible light irradiation. It also achieved a NO removal rate exceeding that of commercial P25 (TiO_2_), a widely used photocatalyst. WP@NC nanocomposites were loaded onto thin g-C_3_N_4_ nanosheets (CN) to create a photocatalyst. This combination significantly increased hydrogen generation compared to pure g-C_3_N_4_ [21]. The N-doped carbon layer enhances the separation and transfer of charge carriers, leading to better stability and corrosion resistance. The CN/WP@NC nanocomposite effectively removed 4-nitrophenol (4-NP) from water under visible light irradiation. The g-C_3_N_4_/NiCNTs composite produced H_2_ at a significantly higher rate than pure g-C_3_N_4_ and even surpasses Pt-based catalysts [22]. The composite efficiently removed harmful 4-nitrophenol (4-NP) under visible light irradiation. Research on g–C_3_N_4_–based sensors for heavy metal detection is an ongoing field with continuous advancements. By optimizing the material properties and sensor design, researchers aim to achieve even higher sensitivity, selectivity, and wider detection ranges. Additionally, integrating g-C_3_N_4_ sensors with portable devices and microfluidic chips holds promise for on-site and real-time environmental monitoring in the future [23].

The aim of the present work is to develop a fluorescence sensor based on magnesium/S@g-C_3_N_4_ for detecting Cu^2+^ ions. S@g-C_3_N_4_ and Mg/S@g-C_3_N_4_ nanosheets were prepared by the polycondensation technique. Polycondensation is a common technique used in materials science to create polymers by linking together smaller molecules. In the case of g-C_3_N_4_, the starting material likely undergoes a chemical reaction at high temperature, causing the small molecules to condense and form the two-dimensional sheets with a specific structure [24]. The presence of magnesium and sulfur suggests they are incorporated into the S@g-C_3_N_4_ framework, potentially modifying its properties for sensing applications. XRD and ATR-FTIR spectroscopy analysis showed the characteristic peaks for S@g-C_3_N_4_ and confirmed that Mg^2+^ incorporation disrupted the regular stacking of tri-*s*-triazine units, potentially leading to increased active sites for enhanced sensing activity. SEM micrograph showed nanosheets with flake-like structures, and EDS analysis confirmed the presence of Mg, S, C, and N, matching the intended composition. The surface area analysis indicated a mesoporous material with a high surface area (40 m^2^/g). The band gap of the Mg/S@g-C_3_N_4_ nanosheet was estimated to be 3.0 eV. The fluorescence intensity of Mg/S@g-C_3_N_4_ decreased as the concentration of Cu^2+^ increased from 1 μM to 20 μM. The sensor offered high sensitivity and selectivity for Cu^2+^ detection over other heavy metals.

## 2 Experimental details

Magnesium chloride hexahydrate (MgCl_2_.6H_2_O, 98 %), thiourea (SC(NH_2_)_2_, 98 %), cadmium chloride anhydrous (CdCl_2_, 99 %), ferric chloride hexahydrate (FeCl_3_.6H_2_O, 97 %), nickel chloride hexahydrate (NiCl_2_·6H_2_O, 98 %), and cobalt (II) chloride hexahydrate (CoCl_2_·6H_2_O, 98 %) were provided by Loba Chemi, India. Copper (II) chloride dihydrate (CuCl_2_·2H_2_O, 99 %) was supplied by Scharlau, Spain. Sodium chloride (NaCl, ≥99.0 %) and calcium chloride (CaCl2, ≥99.0 %) were provided by Sigma-Aldrich, Germany.

The preparation of magnesium/S@g-C_3_N_4_ nanosheets via the polycondensation method involves the thermal decomposition of magnesium chloride (MgCl_2_) and thiourea (SC(NH_2_)_2_) at 550 °C for 2 h. 6.0 mg of magnesium chloride and 12.5 g of thiourea were ground properly to form the desired precursor complex. The obtained powder was put in a clean porcelain crucible suitable for high-temperature reactions. The crucible was placed in a preheated muffle furnace at 550 °C for 2 h with a heating rate of 3 °C/min. This high temperature promotes the decomposition of the precursor and the formation of the magnesium/S@g-C_3_N_4_ nanosheets. Then the furnace was allowed to cool down naturally to room temperature. Finally, the produced yellow powder was ground and applied for experimental testing.

A concentration of 1 mg/mL of Mg/S@g-C_3_N_4_ was prepared. 50 mg of solid Mg/S@g-C_3_N_4_ was dissolved in 50 mL of water. 10 μL of the Mg/S@g-C_3_N_4_ stock solution was diluted with 2 mL of water. This creates a working solution with a lower concentration of Mg/S@g-C_3_N_4_. Different volumes of a 2 μM Cu^2+^ solution was added to the working solution. The mixtures are kept for 5 min under static conditions to allow for equilibration. After equilibration, the fluorescence spectra of the mixtures containing different amounts of Cu^2+^ are recorded. A wavelength of 360 nm was used to excite the Mg/S@g-C_3_N_4_ at room temperature. Finally, the selectivity of the Mg/S@g-C_3_N_4_ towards Cu^2+^ is compared with other metal ions: Cd^2^⁺, Fe^3+^, Ni^2+^, Na^+^, Ca^2+^, and Co^2+^. All the tested metal ions have the same concentration (10 μM) as Cu^2+^. The experiments were conducted at room temperature.

The crystal structure and phase composition of the S@g-C_3_N_4_ and magnesium/S@g-C_3_N_4_ were determined by XRD analysis, which was completed on a Shimadzu XRD 7000 utilizing a Cu_kα_ wavelength of 1.54056 Å. The ATR-FTIR spectra of S@g-C_3_N_4_ and Mg/S@g-C_3_N_4_ were recorded using a Shimadzu 100-FTIR tracer. The information about the morphology and elemental composition was provided by Thermo Fisher Quatro S ESEM electron microscopy equipped with the Stanford energy-dispersive spectroscopy (EDS) Unit. The surface area and porosity of the nanosheet were determined using a NOVA 4200e surface area analyzer. The samples were degassed at 150 °C for 24 h. The UV–Vis spectrum of Mg/S@g-C_3_N_4_ was obtained using a Thermo Scientific Evo 201 spectrophotometer. The fluorescence data of the Mg/S@g-C_3_N_4_ was measured at an excitation wavelength of 360 nm using a Cary Eclipse fluorescence spectrometer.

## 3 Results and discussion

**Fig. 1:**
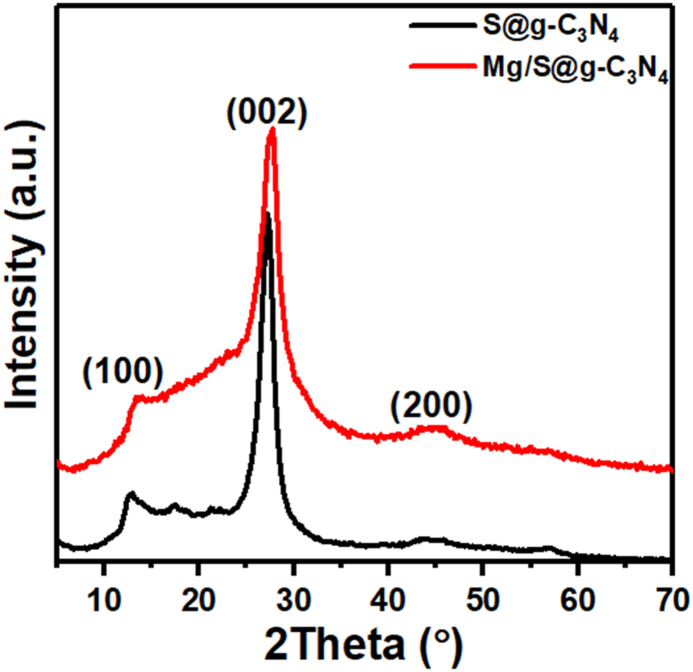
XRD spectrua of S@g-C_3_N_4_ and magnesium/S@g-C_3_N_4_ nanosheet.

The XRD spectrum of the S@g-C_3_N_4_ and magnesium/S@g-C_3_N_4_ nanosheet were plotted in Fig. 1. The characteristic peaks for S@g-C_3_N_4_ are typically observed around 13.0° and 27.4°, corresponding to the (100) and (002) planes, respectively [25,26]. The peak at 27.4° is a characteristic peak for g-C_3_N_4_, and its presence confirms the successful formation of the material. This peak corresponds to the stacking of tri-*s*-triazine units [27]. The absence of diffraction peaks in the XRD spectrum after incorporating magnesium ions into the S@g-C_3_N_4_ framework can be attributed to the dispersion of Mg^2+^ within the S@g-C_3_N_4_ framework, leading to a low concentration in specific crystallographic sites. The broadening of the characteristic peak for S@g-C_3_N_4_ at 27.5° after the addition of Mg^2^⁺ ions suggest several potential effects on the structure: Incorporation of Mg^2^⁺ ions into the S@g-C_3_N_4_ framework can introduce strain and defects, disrupting the long-range order of the crystal structure. This disruption leads to a decrease in the crystallite size and a broadening of the diffraction peaks. Meanwhile, Mg^2^⁺ ions interact with the nitrogen atoms in the tri-*s*-triazine units, pulling them closer and reducing the distance between the S@g-C_3_N_4_ layers. This contraction results in a shift of the (002) peak to a lower angle and a broadening of these peaks. In this context, the interplanar spacing value was estimated for this peak to be 3.26 Å, which is lower than that reported for S@g-C_3_N_4_ [28]. Moreover, a broad peak was observed at 44.6°, which corresponds to the (200) plane of the S@g-C_3_N_4_ crystal structure [29]. This broad peak indicates the presence of an amorphous phase within the S@g-C_3_N_4_ composite. Hence, amorphous materials lack long-range order in their atomic arrangement, leading to broad and diffuse peaks in the XRD spectrum. Accordingly, the incorporation of Mg^2^⁺ ions into the S@g-C_3_N_4_ framework disrupt the regular stacking of tri-*s*-triazine units, leading to stacking disorder. Stacking disorder can induce more active sites on the surface of the S@g-C_3_N_4_ due to exposed edges and defect sites. These sites can facilitate the adsorption of reactant molecules, potentially leading to higher fluorescence sensing activity [30].

Crystallite size refers to the dimensions of the individual crystalline domains within a material. The Scherrer equation provides a means to estimate this size (D) based on the peak broadening (β) observed during X-ray diffraction experiments [31,32].

(1) $$D=\frac{0.9\lambda }{\beta \;\cos\;\theta }$$

where λ is the X-ray wavelength and θ is the peak position in degrees. The peak broadening of S@g-C_3_N_4_ sheets was found to be 0.0326 radians. In the case of Mg/S@g-C_3_N_4_ nanosheets, we can use this equation to estimate the sheet size based on the broadening of the peak at 27.4°. The broad full width at half maximum (0.056 radians) implies smaller crystallite size, representing smaller S@g-C_3_N_4_ sheets. Smaller sheets provide a larger surface area per unit volume, offering more sites for analyte interaction and adsorption. This increased surface area allows for more efficient capture of target molecules, leading to a stronger fluorescence response.

**Fig. 2:**
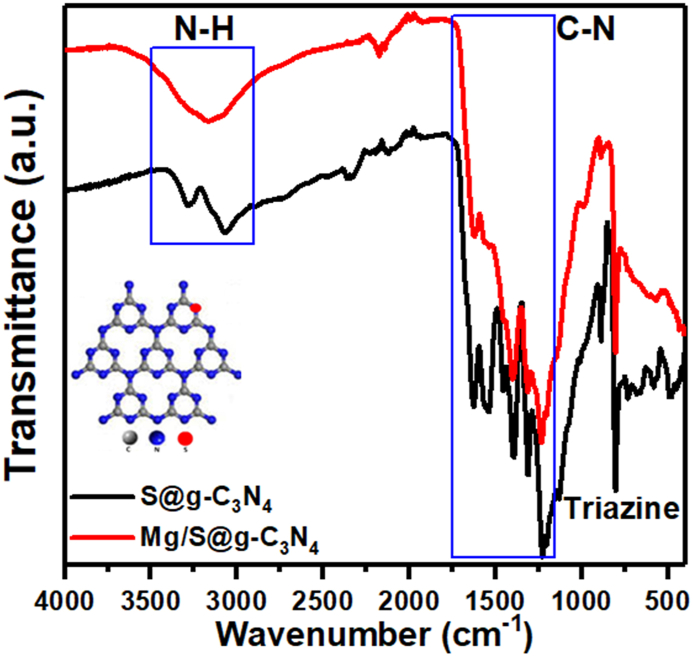
ATR-FTIR spectra of S@g-C_3_N_4_ and magnesium/S@g-C_3_N_4_ nanosheet.

The ATR-FTIR spectraof the S@g-C_3_N_4_ and magnesium/S@g-C_3_N_4_ nanosheet is given in Fig. 2. A strong peak around 804 cm^−1^ indicates the presence of triazine units, the building blocks of g-C_3_N_4_. This peak is associated with the breathing mode of the triazine ring, and its presence confirms the successful formation of the g-C_3_N_4_ structure. Moreover, indicates that the basic g-C_3_N_4_ framework remains intact even after incorporating Mg^2^⁺ and sulfur atoms, suggesting that the material retains the potential properties of g-C_3_N_4_, such as fluorescence sensing activity and high thermal stability [33].

Peaks between 1200 and 1700 cm⁻^1^ correspond to the stretching vibrations of aromatic CN bonds. The aromatic C–N stretching vibrations in triazine units give the bands within 1200–1350 cm^−1^. The conjugated C=N stretching reveals peaks within 1400–1600 cm^−1^. The presence of additional peaks, such as the amide I band at 1550-1650 cm^−1^ or the C=O stretching at 1660-1700 cm^−1^, is an indicative of functional groups other than CN bonds in the material [34]. Analyzing the relative intensity of peaks in the 1400-1600 cm^−1^ and 1200-1350 cm^−1^ regions in the ATR-FTIR spectrum of Mg/S@g-C_3_N_4_ offers a valuable tool for assessing the degree of conjugation within the framework. From inspection of Fig. 2, the relative intensity of peaks in the 1400-1600 cm^−1^ is lower than that of 1200–1350 cm^−1^ region. Therefore, a lower intensity ratio between the two regions indicates a lower degree of conjugation within the Mg/S@g-C_3_N_4_ framework. This can be attributed to the incorporation of Mg^2^⁺ ions, and sulfur atoms can disrupt the regular arrangement of tri-*s*-triazine units in the g-C_3_N_4_ framework. This disruption can introduce defects and imperfections, interrupting the delocalization of electrons along the conjugated network. As a result, the number of C=N bonds with conjugated double bonds decreases, leading to a decrease in the intensity of the peak in the 1400-1600 cm^−1^ region [35]. A broad peak observed in the 3100-3600 cm^−1^ region of the ATR spectrum can be indicative of the presence of free amino groups (-NH_2_) in the Mg/S@g-C_3_N_4_. This peak is associated with the stretching vibrations of the N–H bond in the amino group [36].

**Fig. 3:**
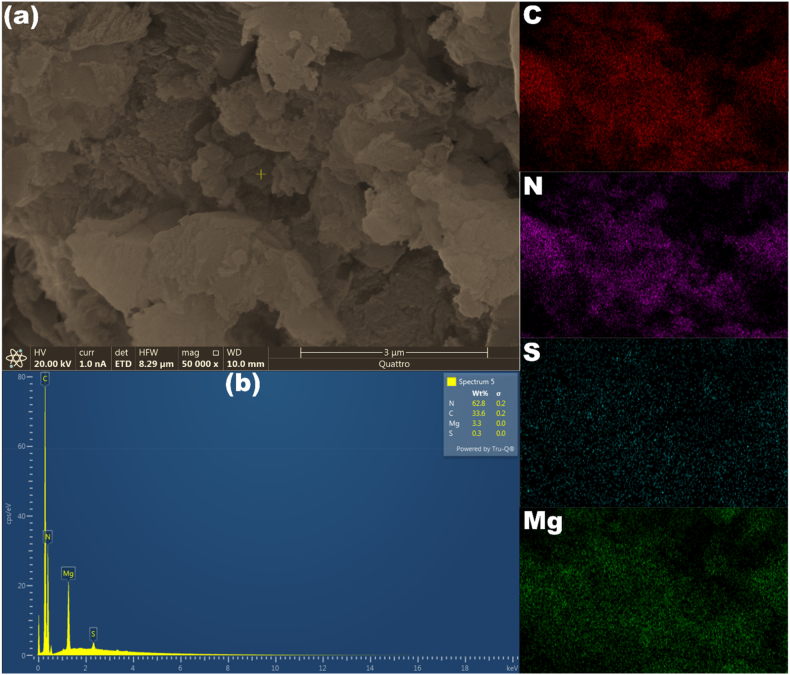
The scans of (a) SEM, (b) EDS and elemental mapping for magnesium/S@g-C_3_N_4_ nanosheet.

Fig. 3a shows the SEM image of a magnesium/S@g-C_3_N_4_ nanosheet. The image shows flakes or sheet-like structures, which are consistent with the morphology of non-exfoliated nanosheets. The EDS analysis displayed in Fig. 3b confirmed the presence of magnesium (Mg), sulfur (S), carbon (C), and nitrogen (N) in the magnesium/S@g-C_3_N_4_ nanosheets, which is consistent with its intended composition.

The EDS mapping shown in Fig. 3 confirmed the presence of magnesium and sulfur throughout the nanosheets, which is consistent with the intended composition of magnesium/S@g-C_3_N_4_ nanosheets.

**Fig. 4:**
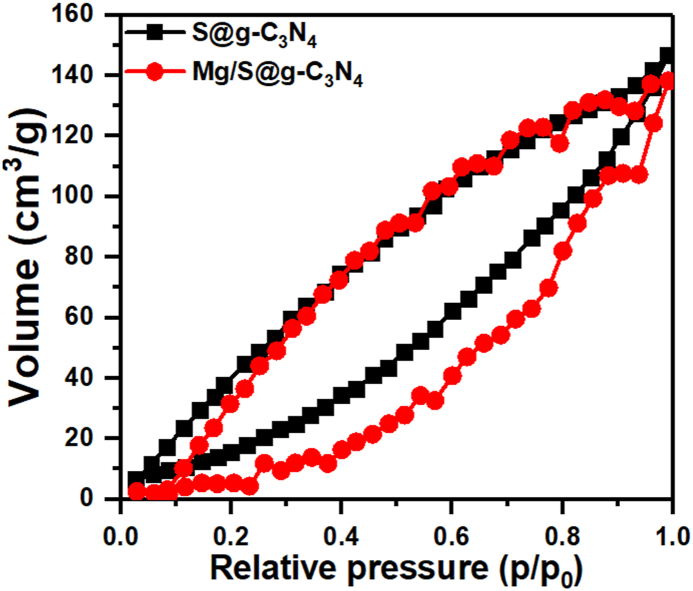
N_2_ isotherms of S@g-C_3_N_4_ and magnesium/S@g-C_3_N_4_ nanosheet.

Fig. 4 displays the N_2_ isotherms of the S@g-C_3_N_4_ and magnesium/S@g-C_3_N_4_ nanosheet. The N_2_ isotherms for S@g-C_3_N_4_ and magnesium/S@g-C_3_N_4_ nanosheets is consistent with a mesoporous material with a high surface area. The BET surface area of 40 m^2^/g was determined for Mg/S@g-C_3_N_4_ nanosheets. A BET surface area of 40 m^2^/g signifies a relatively high surface area for this material. This suggests the presence of numerous pores and a good potential for applications that involve surface interactions, like catalysis or adsorption. This provides ample space for heavy metal ions to interact with the surface, enhancing the sensitivity of the sensor [37].

Mesoporous materials have pores that are less than 2 nm in diameter. In this context, the BJH pore sizes of the S@g-C_3_N_4_ and Mg/S@g-C_3_N_4_ nanosheet were determined to be 2.0 and 1.57 nm. The BJH pore size of 1.57 nm falls within the range suitable for capturing heavy metal ions. Many heavy metal ions have ionic radii around 0.5–1.0 nm, so the mesopores in the nanosheets can potentially act as sieves, allowing heavy metal ions to enter while excluding larger molecules.

**Fig. 5:**
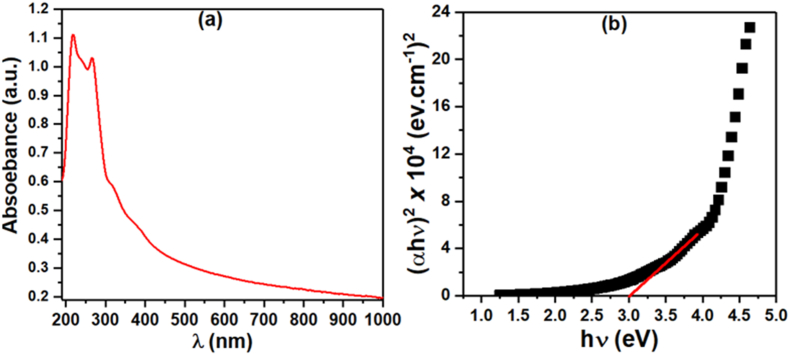
The plots of (a) absorbance versus wavelength and (b) Tauc plot for magnesium/S@g-C_3_N_4_.

The optical absorption spectrum of the Mg/S@g-C_3_N_4_ nanosheet is given in Fig. 5a. The absorption onset is around 410 nm. This corresponds to an energy of approximately 3.0 eV. The material shows broad absorption throughout the ultraviolet (UV) and visible light ranges (200 nm–700 nm). This suggests that magnesium/S@g-C_3_N_4_ can absorb a wide range of wavelengths within this region. When Mg substitutes for carbon atoms in the S@g-C_3_N_4_ lattice, it creates a positive charge imbalance. This can alter the electronic structure and push the valence and conduction bands further apart, resulting in a wider band gap.

Fluorescence sensors, reliant on the ability of materials to absorb light and re-emit it at a different wavelength, play a crucial role in various analytical applications. However, not all materials are created equal for this purpose. The band gap, a fundamental property of a material, significantly influences a sensor's performance, impacting its excitation efficiency, emission wavelength, and even selectivity. An effective sensor needs to be efficiently excited by the chosen light source. The band gap dictates the wavelength of light required for excitation. A material with a band gap that aligns well with the excitation source will absorb light more effectively, resulting in brighter fluorescence. This translates to a stronger signal and ultimately better sensitivity. The Tauc plot method is a widely used technique to estimate the band gap energy (E_g_) of semiconductor materials using UV–Vis absorption spectroscopy data. If the momentum of an electron in the valence band is equal to the momentum of the excited electron in the conduction band, it's a direct transition. For direct band gap materials, the Tauc relation is [38,39]:

(2) $${\left(\alpha h\nu \right)}^{2}=A\left(h\nu \;-\;E_{g}\right)$$

where α is the absorption coefficient and A is a constant. The data of (αhν)^2^ was plotted on the y-axis and the photon energy (hν) on the x-axis, as shown in Fig. 5b. The x-intercept of the extrapolated linear regression line on the energy axis (hν) represents the band gap energy (E_g_) of the material. The band gap of the Mg/S@g-C_3_N_4_ nanosheet was estimated to be 3.0 eV. A wider band gap (3.0 eV) in magnesium/S@g-C_3_N_4_ compared to S@g-C_3_N_4_ (2.4 eV) [40] could potentially lead to improved selectivity for specific heavy metals. Heavy metal ions can interact with the sensor material and create new energy levels within the band gap. If the band gap of the sensor is wider, it might require a more specific energy input from a heavy metal ion to reach an excited state, potentially leading to a more selective response for certain metal ions [41].

**Fig. 6:**
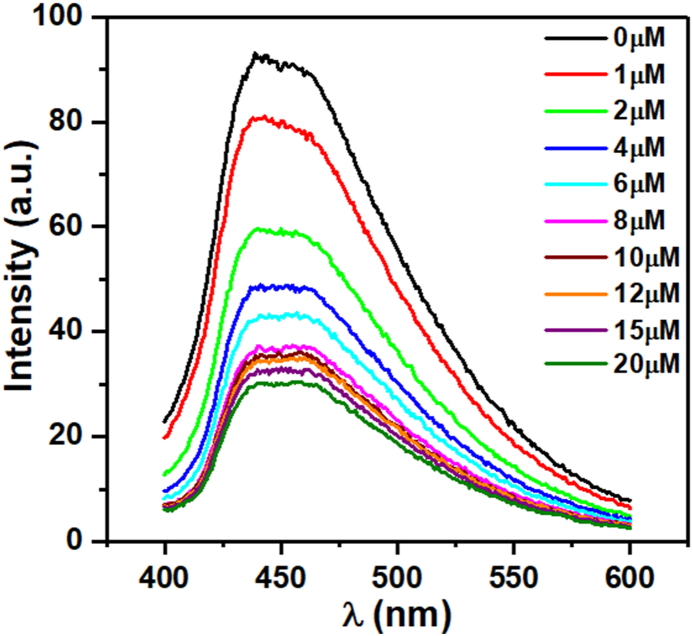
Fluorescence spectra of Mg/S@g-C_3_N_4_ at different concentration of Cu^2+^ (λ_exc_ = 360 nm).

Fig. 6 shows the fluorescence intensity of Mg/S@g-C_3_N_4_ at different concentrations of Cu^2+^. The graph shows a linear relationship between the fluorescence intensity and the concentration of Cu^2+^. This means that the fluorescence intensity decreases linearly as the concentration of Cu^2+^ increases.

The fluorescence intensity of Mg/S@g-C_3_N_4_ decreases as the concentration of Cu^2+^ increases, which is a phenomenon known as quenching. This quenching effect is because Cu^2+^ ions can act as electron acceptors, which can steal electrons from the excited state of Mg/S@g-C_3_N_4_ molecules. As a result, the Mg/S@g-C_3_N_4_ molecules are unable to emit light, leading to a decrease in fluorescence intensity [42]. The concentration of Cu^2+^ plays a role here. With more Cu^2+^ ions around, there are more "thieves" to steal electrons, leading to a more significant decrease in fluorescence intensity, as observed in Fig. 6. The quenching effect can be used to develop sensors for Cu^2+^ ions. In this case, the fluorescence intensity of Mg/S@g-C_3_N_4_ can be used to measure the concentration of Cu^2+^ ions in a sample.

Quenching mechanisms describe how excited molecules lose their energy before they can emit light. In static quenching, the excited molecule and the quencher form a complex together before any excitation happens. Because they are already stuck together, the excited energy gets shuffled around within the complex and again isn't emitted as light. Dynamic quenching occurs when an excited molecule (fluorophore) bumps into another molecule (quencher) and transfers its energy to the quencher in a collision. The quencher dissipates this energy in ways that don't involve light emission. Combined dynamic and static quenching involves a bit of both. The quencher can collide with the molecule (dynamic quenching) and form complexes with it (static quenching). This can be especially effective at quenching fluorescence because there are two different ways for the excited energy to be lost [43]. The ratio I_0_/I (initial intensity divided by final intensity) relate to the concentration of the quencher. In cases of dynamic and static quenching, this relationship is linear. This means as the concentration of the quencher increases, the ratio F_0_/F also increase in a straight-line fashion when plotted on a graph. K_SV_ is a constant called the Stern-Volmer quenching constant. It provides information about how efficiently the quencher quenches the fluorescence. The steeper the slope of the linear relation between F_0_/F and quencher concentration, the higher the K_SV_ value. This means a steeper slope indicates a more efficient quencher. The Stern-Volmer equation describes the relationship between the intensity of fluorescence (emitted light) and the concentration of a quencher molecule in a system [44,45].

(3) $$\frac{F_{0}}{F}=1+K_{SV}\left[{Cu}^{2+}\right]$$

**Fig. 7:**
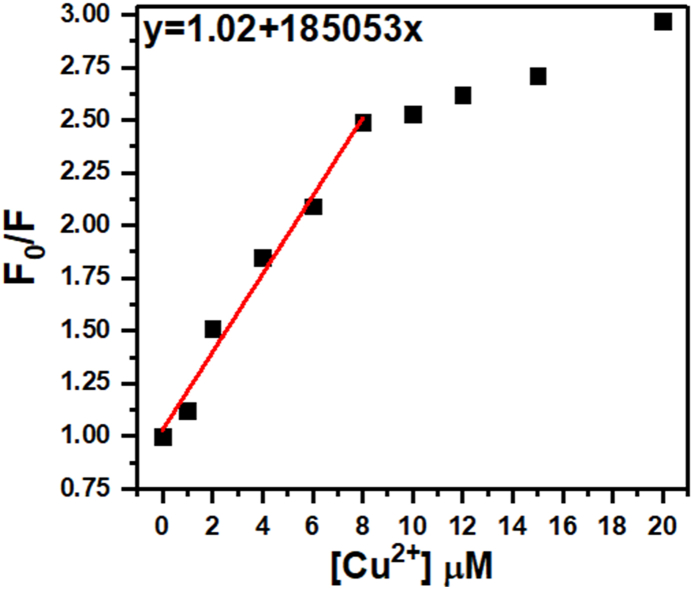
Plot of F_0_/F versus the concentration of the quencher [Cu^2+^].

The plot of F_0_/F versus the concentration of the quencher is given in Fig. 7. At low quencher concentrations, there are fewer quencher molecules around. This means the excited fluorophore molecules have a good chance of emitting light before encountering a quencher. The quenching process is dominated by random collisions between the fluorophore and quencher, and the rate of quenching increases proportionally with quencher concentration. This results in a linear relationship on the Stern-Volmer plot. However, at high quencher concentrations, the quenching process becomes saturated. Not all collisions lead to quenching because some fluorophores are quenched so quickly, they can't even bump into another quencher molecule. This saturation effect causes the curve to deviate from linearity and typically bend upwards on the Stern-Volmer plot. Therefore, we have used the quenching data obtained within the linear range of the Stern-Volmer plot (0–8 μM in this case) to calculate the Stern-Volmer quenching constant (K_SV_). Accordingly, the relatively high K_SV_ (185053 M^−1^) suggests that the quencher molecule is quite effective in reducing the fluorescence of the fluorophore at the concentrations used in the analysis.

The LOD (limit of detection) for Cu^2+^ ions were estimated based on a signal to noise ratio of 3.3, via the equation: LOD = 3.3(δ_blank_/K_SV_) [46], where δ_blank_ is the standard deviation of the blank solution. The estimated value of LOD for Cu^2+^ was 16.2 nM. Our Cu^2+^ sensor's LOD appears promising compared to most of the previously reported sensors [[47], [48], [49], [50]], and [51].

g-C_3_N_4_ presented a promising avenue for developing new, efficient, and cost-effective methods for metal ion detection as shown in Table 1.

**Table 1:** The detection of metal ions using g-C_3_N_4_ sensor.

Metal ion	Linear range	LOD (nM)	Ref.
Ag^+^	0–40 nmol/L	4.2	[52]
Au^3+^	0–15 μM	1100	[53]
Fe^3+^	0–100 μM	8700	[54]
Hg^2+^	0–60 μM	620	[55]
Cr^6+^	1–100 μM	300	[56]
Cu^2+^	0–8 μM	16.2	This work

**Fig. 8:**
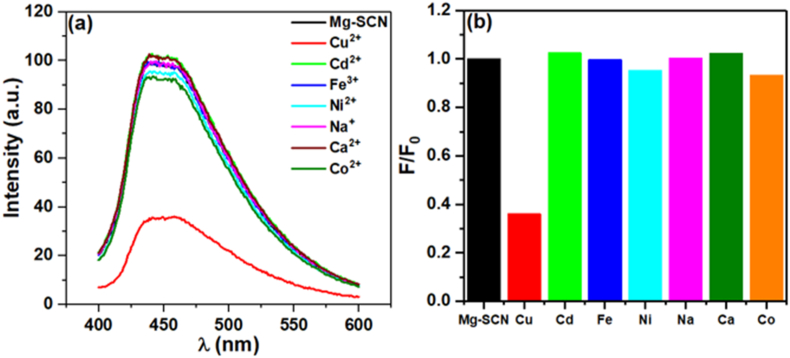
Selectivity test of Mg/S@g-C_3_N_4_ for different heavy metals.

The ratio of the fluorescence intensity (F) of Mg/S@g-C_3_N_4_ in the presence of a specific heavy metal to the initial fluorescence intensity (F_0_) of Mg/S@g-C_3_N_4_ alone (F/F_o_) was plotted in Fig. 8a for different heavy metals. A lower F/F_0_ ratio indicates a greater quenching effect, signifying higher selectivity.

The provided data in Fig. 8b confirms that Mg/S@g-C_3_N_4_ demonstrates selectivity for Cu^2+^ over other heavy metals. Compared to other metals, Cu^2+^ shows the most significant decrease in fluorescence intensity, as indicated by the lowest F/F_0_ ratio on the graph. This implies that Mg/S@g-C_3_N_4_ has the highest selectivity for Cu^2+^ ions among the tested metals. While not as prominent as Cu^2+^, some other metals like Fe and Co also lead to a slight decrease in fluorescence intensity, suggesting a minor quenching effect. However, their F/F_0_ ratios are significantly higher than Cu^2+^, indicating a considerably weaker interaction with Mg/S@g-C_3_N_4_. The F/F_0_ ratios for Na and Ca are very close to 1, indicating a negligible quenching effect. This suggests that Mg/S@g-C_3_N_4_ has minimal interaction with these elements, making it a selective sensor for Cu^2+^ in their presence. The experiment demonstrated that Mg/S@g-C_3_N_4_ nanosheets exhibit a strong quenching response towards Cu^2+^ ions compared to other tested heavy metals. This makes Mg/S@g-C_3_N_4_ a promising candidate for selective Cu^2+^ sensing applications.

## 4 Conclusions

The present work successfully developed a novel fluorescence sensor based on Mg/S@g-C_3_N_4_ nanosheets for selective detection of Cu^2+^ ions in water. Mg/S@g-C_3_N_4_ nanosheets were prepared by the polycondensation technique and investigated by XRD, ATR-FTIR spectroscopy, SEM, surface area, and UV–Vis optical absorption measurements. XRD analysis confirmed the successful formation of Mg/S@g-C_3_N_4_ and revealed the potential disruption of the regular stacking of tri-*s*-triazine units due to Mg^2+^ incorporation. ATR-FTIR analysis confirmed the presence of key functional groups and indicated a lower degree of conjugation within the Mg/S@g-C_3_N_4_ framework. SEM analysis revealed the formation of nanosheets with a slightly wrinkled and interconnected morphology. The Mg/S@g-C_3_N_4_ nanosheets exhibited a high surface area (40 m^2^/g). The sensor material possessed a wider band gap of 3.0 eV. The fluorescence intensity of Mg/S@g-C_3_N_4_ nanosheets exhibited a linear decrease with increasing Cu^2+^ concentration, indicating high sensitivity. The estimated value of LOD by our sensor for Cu^2+^ was 16.2 nM. Furthermore, the sensor displayed remarkable selectivity for Cu^2+^ detection compared to other tested metal ions. These findings suggest that Mg/S@g-C_3_N_4_ nanosheets hold great promise as a viable sensing platform for the selective and sensitive detection of Cu^2+^ ions in water samples. This research paves the way for further advancements in environmental monitoring and water quality control, offering a potential solution for the detection of harmful heavy metal contaminants.

## Research data policy and data availability statements

Data will be available upon author request.

## CRediT authorship contribution statement

**Z.A. Alrowaili:** Methodology, Funding acquisition, Formal analysis, Data curation, Conceptualization. **Asmaa I. El-Tantawy:** Validation, Resources, Investigation. **S.A. Saad:** Validation, Investigation, Conceptualization. **M.H. Mahmoud:** Validation, Supervision, Resources, Data curation. **Karam S. El-Nasser:** Methodology, Formal analysis, Data curation, Conceptualization. **Taha Abdel Mohaymen Taha:** Writing – original draft, Supervision, Methodology, Investigation, Formal analysis, Data curation, Conceptualization.

## Declaration of competing interest

The authors declare that they have no known competing financial interests or personal relationships that could have appeared to influence the work reported in this paper.
